# A Rare Case of *Burkholderia cepacia* Complex Septic Arthritis

**DOI:** 10.1155/2018/6232760

**Published:** 2018-09-16

**Authors:** John Koo, Gregory D. Deans

**Affiliations:** ^1^Department of Medicine, University of British Columbia, Vancouver, BC, Canada; ^2^Section of Infectious Diseases, Department of Medicine, Surrey Memorial Hospital, Surrey, BC, Canada

## Abstract

Bacteria of the *Burkholderia cepacia* complex have rarely been reported to cause septic arthritis. Cases have been reported in patients who were immunocompromised, at extremes of age or who had history of steroid injection or penetrating trauma. A 67-year-old man with a history of opioid use disorder, osteoarthritis, and gout but no known immunocompromise was admitted to hospital with pain and swelling of his right knee. Cultures of synovial fluid and urine grew *Burkholderia cepacia* complex. Microscopy of synovial fluid also identified intracellular calcium pyrophosphate crystals. The patient's symptoms improved with joint irrigation and debridement and prolonged antimicrobial therapy. This case highlights the importance of diagnostic aspiration of an acutely inflamed joint to obtain a specific etiological diagnosis.

## 1. Introduction

*Burkholderia cepacia*, a Gram-negative aerobic bacillus formerly known as *Pseudomonas cepacia*, was first identified in 1950 from the rot of onion bulbs [1]. *B. cepacia* complex is composed of closely related species of bacteria found ubiquitously in nature. Organisms from this complex are well-known opportunistic pathogens of patients with cystic fibrosis, lung transplantation, and chronic granulomatous disease. However, joint infections due to organisms of the *B. cepacia* complex are rare with few reported cases.

## 2. Case Presentation

A 67-year-old man was brought to the emergency department by ambulance after being found lying on the floor of his home. He reported a fall onto his right hip with a long lie for an estimated 24 hours. His medical history was significant for opioid use disorder, gout, hypertension, and osteoarthritis. He had undergone prosthetic joint replacement of the left shoulder nine years before this presentation. He had chronic bilateral knee pain but reported acute worsening of pain in his right knee starting one day before the fall. He endorsed a history of intramuscular injection of opioids to his buttocks and upper arms but denied injection to his hip or knee.

**Figure 1:**
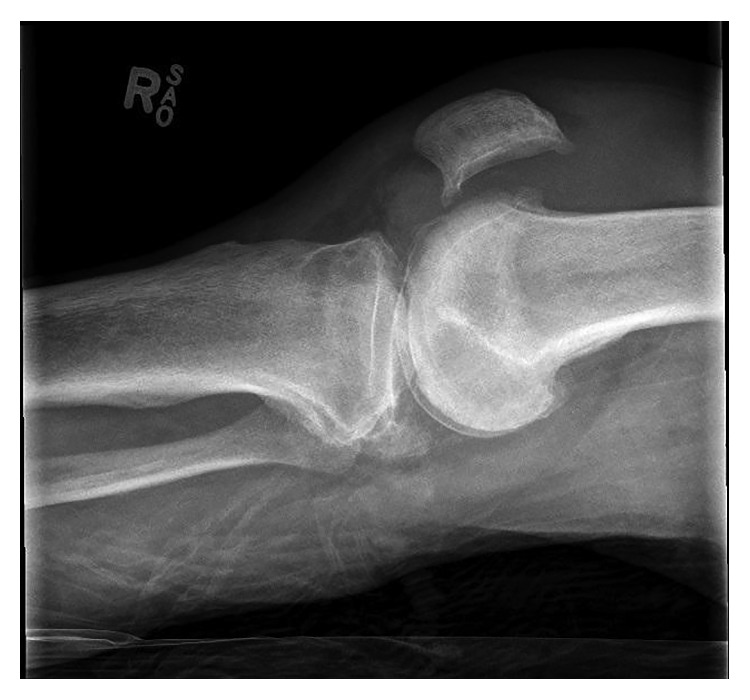
X-ray of the patient's right knee on the day of presentation shows soft tissue swelling but no discrete fracture. There is tricompartmental osteoarthritis with a moderately large joint effusion.

On examination, he was found to have a swollen right knee with no erythema or signs of trauma. The X-ray of the right knee is shown in Figure 1. His initial blood pressure was 240/120 mmHg. His serum creatinine of 159 *µ*mol/L was significantly increased from baseline, but creatine kinase was not markedly elevated (447 U/L). Laboratory testing also revealed leukocytosis (20.2 × 10^9^ cells/L) with neutrophil predominance (18.2 × 10^9^ cells/L) as well as elevated C-reactive protein (292 mg/L). Serological tests for HIV, hepatitis B, and hepatitis C were negative.

After admission to hospital, the patient's kidney injury resolved promptly with volume repletion, and his hypertension responded to antihypertensive medication. On his third day in hospital, the patient reported urinary urgency without dysuria or gross hematuria, which prompted investigation though he later denied having had urinary symptoms. Urine dipstick showed positive blood (250 ery/*µ*L), positive leukocyte esterase, and positive nitrite; according to local laboratory protocol, microscopy of urine sediment and Gram stain were not performed. Urine culture showed >100 × 10^6^ CFU/L yellow-grey colonies and nonlactose fermenting colonies on blood agar and MacConkey agar, respectively, after 24 hours of incubation. Oxidase test was positive. It was identified as *B. vietnamiensis*, a *B. cepacia* complex organism, by BD™ Bruker MALDI Biotyper™.

During his admission, the patient's right knee became more swollen and painful until he could no longer mobilize. Treatment for gout was ineffective in relieving his symptoms, so a joint aspiration of the right knee was performed. The knee joint aspirate was frankly purulent fluid with a nucleated cell count of 26 766 × 10^6^ cells/L, of which 95% were neutrophils, and microscopy revealed intracellular calcium pyrophosphate crystals. Culture grew >100 × 10^6^ CFU/L grey colonies on blood and chocolate agar plates after 24 hours of incubation. The Gram stain from the growth showed Gram-negative rods, which were also identified as *B. vietnamiensis* by MALDI. For the synovial fluid isolate, susceptibility testing by E-Test® indicated minimum inhibitory concentration values in the susceptible range for meropenem, levofloxacin, trimethoprim/sulfamethoxazole, ceftazidime, and minocycline (0.5 *µ*g/mL, 1 *µ*g/mL, 0.75–1 *µ*g/mL, 0.75–1 *µ*g/mL, and 0.38–0.5 *µ*g/mL, respectively) according to Clinical and Laboratory Standards Institute breakpoints [2]. Antibiotic susceptibility profile for the urine culture isolate using Vitek 2® was similar to the results from the knee joint aspirate. Blood cultures had no growth of bacteria. Transthoracic echocardiogram showed no evidence of endocarditis.

The patient was treated with three days of empiric meropenem, which was subsequently switched to ceftazidime with a plan to complete six weeks of therapy. Irrigation and debridement of the knee joint was performed four days after the diagnostic aspiration. His knee pain improved significantly, and after twenty-eight days of antibiotic therapy, the patient left the hospital against medical advice. By the time of discharge, the patient's leukocyte and C-reactive protein levels had normalized.

## 3. Discussion

Septic arthritis caused by *B. cepacia* complex is rarely reported. Most previous case reports involved patients who were immunocompromised, at extremes of age or who had history of steroid injection or penetrating trauma. The first reported case occurred in an immunocompromised patient who experienced a spontaneous infection after allogeneic stem cell transplant for angioimmunoblastic T-cell lymphoma [3]. After surviving four episodes of *B. cepacia* bacteremia, the patient developed septic arthritis of the shoulder and a fifth episode of bacteremia with the organism; despite antibiotic therapy and arthroscopic surgery, the patient ultimately succumbed to the infection. Subsequently, joint infection has been reported in a 3-month-old preterm infant with bilateral hip joint infection [4]; an elderly patient with total dependence for activities of daily living who developed *B. cepacia* infection in the knee [5]; and a young healthy man who had a penetrating trauma to the knee complicated by infection with multiple Gram-negative bacilli including *B. cepacia* [6]. Iatrogenic infection following joint injection has also been described [7] including one report in which *B. cepacia* contamination of a multidose vial of corticosteroid was confirmed [8].

Our case of *B. cepacia* complex septic arthritis in a man with no known compromise of his immune system likely represents bacteremic spread associated with his history of intramuscular self-injection of opioids. The patient adamantly denied injection near his knee, making direct inoculation less likely. However, drug injection can also cause bacteremia or metastatic infection with organisms from the commensal flora or from microbial contamination of drugs, drug use equipment, and the injection environment [9]. *B. cepacia* has been reported as a cause of infective endocarditis in heroin users [10]. Our patient's transthoracic echocardiogram did not show evidence of endocarditis, but the growth of *B. cepacia* complex organisms with identical susceptibility patterns from synovial fluid and urine indicates that the likely mechanism for his infection was transient bacteremia with hematogenous spread.

Our patient may have been predisposed to septic arthritis by the intracellular calcium pyrophosphate crystals found in his synovial fluid as well as his history of osteoarthritis and gout, although we could not find evidence of a definitive diagnosis of gout in the past. Many patients with septic arthritis have preexisting joint disease [11]. Cases of concomitant septic arthritis and crystal arthritis have been reported as well, with higher synovial leukocyte counts seen in patients with concomitant disease than in those with crystal arthritis only [12]. Although the mechanism is unclear, one theory is that crystal-induced damage predisposes a joint to infection. Alternatively, inflammation from joint infection may release crystals from the synovial membrane. Septic arthritis is also associated with prosthetic joints, but our patient did not have any signs of infection in his prosthetic shoulder joint.

Treatment of septic arthritis requires antimicrobial therapy and joint drainage. As there have been no randomized controlled trials comparing antibiotic regimens, initial therapy is based on clinical presentation and Gram stain results [13]. In our case, joint infection was not suspected until joint fluid culture grew Gram-negative bacteria, at which time empiric treatment with meropenem was initiated due to the possibility of infection with *Pseudomonas* species among injection drug users [14]. Septic arthritis with a Gram-negative organism is usually treated with parenteral therapy for the full duration unless the isolate is susceptible to an appropriate oral fluoroquinolone, in which case completing the latter part of therapy orally may be reasonable. In our case, we were concerned about the patient's potential adherence to oral therapy with levofloxacin and instead selected ceftazidime for his definitive treatment.

Treatment of *B. cepacia* complex organisms may prove to be challenging as they are known to exhibit multidrug resistance due to innate and acquired mechanisms including adherence to epithelial cells, biofilm formation, and secretion of factors that help to evade host defenses [15]. Antimicrobials used for the empiric therapy of septic arthritis may not be active against this organism. There is also a lack of trial evidence to guide the optimal antibiotic regimen for treatment of *B. cepacia* complex infections even for patients with cystic fibrosis among whom *B. cepacia* complex organisms are commonly encountered [15]. Therefore, appropriate therapy requires early recognition and antibiotic susceptibility profile.

## 4. Conclusion

Our report of a case of septic arthritis of the knee with *B. cepacia* complex in a patient thought initially to have a flare of gout highlights the importance of diagnostic aspiration of an acutely inflamed joint. Appropriate synovial fluid analysis including a Gram stain and culture besides other relevant tests can provide a specific etiological diagnosis and help in evaluation of a possible concurrent infectious process. The possibility of an infectious process must always be borne in mind. A history of injection drug use in the context of septic arthritis should raise suspicion for hematogenous spread. When infection is present, the results of culture and susceptibility testing inform appropriate medical decision-making regarding source control and antimicrobial prescribing.

## References

[1] Burkholder W. (1950). Sour skin, a bacterial rot of onion bulbs. *Phytopathology*.

[2] Clinical and Laboratory Standards Institute (CLSI) (2018). *Performance Standards for Antimicrobial Susceptibility Testing*.

[3] Miki R. A., Rubin L. E., Kirk J., Dodds S. D. (2006). Spontaneous septic arthritis caused by *Burkholderia cepacia*. *Iowa Orthopedic Journal*.

[4] Nivedhana S., Sulochana P., Shobana R. (2016). Spontaneous septic arthritis due to Burkholderia cepacia in a 3-month-old pre-term infant. *Indian Journal of Medical Microbiology*.

[5] Rodriguez M. S., de la Fuente J., Montero J., Rodriguez M. I. (2010). Arthritis caused by *Burkholderia cepacia*. *Enfermedades Infecciosas Y Microbiologia Clinica*.

[6] Chiu L. Q., Wang W. (2013). A case of unusual gram-negative bacilli septic arthritis in an immunocompetent patient. *Singapore Medical Journal*.

[7] Matteson E. L., McCune W. J. (1990). Septic arthritis caused by treatment-resistant *Pseudomonas cepacia*. *Annals of Rheumatic Diseases*.

[8] Kothari T., Reyes M. P., Brooks N. (1977). *Pseudomonas cepacia* septic arthritis due to intraarticular injections of methylprednisolone. *Canadian Medical Association Journal*.

[9] Kaushik K. S., Kapila K., Praharaj A. K. (2011). Shooting up: the interface of microbial infections and drug abuse. *Journal of Medical Microbiology*.

[10] Noriega E. R., Rubinstein E., Simberkoff M. S., Rahal J. J. (1975). Subacute and acute endocarditis due to *Pseudomonas cepacia* in heroin addicts. *American Journal of Medicine*.

[11] Kaandorp C. J., Krijnen P., Moens H. J., Habbema J. D., van Schaardenburg D. (1997). The outcome of bacterial arthritis: a prospective community-based study. *Arthritis & Rheumatology*.

[12] Shah K., Spear J., Nathanson L. A., McCauley J., Edlow J. A. (2007). Does the presence of crystal arthritis rule out septic arthritis?. *Journal of Emergency Medicine*.

[13] Sharff K. A., Richards E. P., Townes J. M. (2013). Clinical management of septic arthritis. *Current Rheumatology Reports*.

[14] Allison D. C., Holtom P. D., Patzakis M. J., Zalavras C. G. (2010). Microbiology of bone and joint infections in injecting drug abusers. *Clinical Orthopedics and Related Research*.

[15] Horsley A., Jones A. M., Lord R. (2016). Antibiotic treatment for *Burkholderia cepacia* complex in people with cystic fibrosis experiencing a pulmonary exacerbation. *Cochrane Database of Systematic Reviews*.

